# Micromechanical mode-localized electric current sensor

**DOI:** 10.1038/s41378-022-00375-1

**Published:** 2022-04-14

**Authors:** Han Li, Zhao Zhang, Luhan Zu, Yongcun Hao, Honglong Chang

**Affiliations:** Ministry of Education Key Laboratory of Micro and Nano Systems for Aerospace, School of Mechanical Engineering, Northwestern Polytechnical University, 127 Youyi West Road, Beilin District, Xi’an, 710072 China

**Keywords:** NEMS, Sensors

## Abstract

This paper outlines the design of a novel mode-localized electric current sensor based on a mechanically sensitive element of weakly coupled resonator systems. With the advantage of a high voltage sensitivity of weakly coupled resonator systems, the current under test is converted to voltage via a silicon shunt resistor, which causes stiffness perturbation to one resonator. The mode-localization phenomenon alters the energy distribution in the weakly coupled resonator system. A theoretical model of current sensing is established, and the performance of the current sensor is determined: the sensitivity of the electric current sensor is 567/A, the noise floor is 69.3 nA/√Hz, the resolution is 183.6 nA, and the bias instability is 81.6 nA. The mode-localized electric current sensor provides a new approach for measuring sub-microampere currents for applications in nuclear physics, including for photocurrent signals and transistor leakage currents. It could also become a key component of a portable mode-localized multimeter when combined with a mode-localized voltmeter. In addition, it has the potential for use in studying sensor arrays to achieve higher resolution.

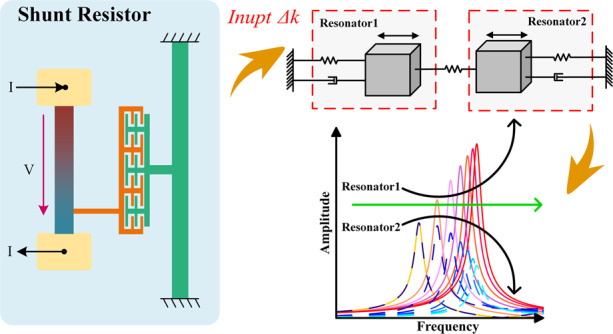

## Introduction

Current is one of the seven fundamental quantities in physics^1^. The precise measurement of current is important in modern scientific research and production^2,3^. With the development of microelectromechanical system (MEMS) sensors, numerous MEMS current sensing techniques and materials have been developed based on various principles, including the Hall effect^4^, magnetic metal film^5^ and giant magnetoresistance^6^. Recently, in the MEMS field, a new transduction scheme based on the mode localization of weakly coupled resonator systems (WCRs) has emerged. Mode localization^7–12^ is a manifestation of Anderson localization^13^ in the field of structural dynamics. It has been shown that mode-localized sensors have high parametric sensitivity and are insensitive to changes in temperature and pressure^14–18^. The mode localization phenomenon can be described as follows: a weak stiffness disturbation breaks the balance of WCRs, resulting in the redistribution of energy, which is manifested as a change in the amplitude ratio (AR). Using this principle, ultrahigh-sensitive mass sensora^18–21^, accelerometers^22–24^, stiffness sensors^25^, electrometers^26,27^, electric field sensors^28,29^, voltmeters^30^ and magnetometers^31^ have been proposed. In particular, a mode-localized voltmeter demonstrated that this principle is suitable for the measurement of electrical quantities. Therefore, we propose a new sensor to sense current using the mode localization phenomenon.

In our previous study^30^, a mode-localized voltmeter was proposed. We believe that the mode-localized electric current sensor (ECS) is a critical part of the development of MEMS multimeters in the future. With the inherent advantages of MEMS devices, several ECSs and voltmeters can be integrated into a single chip. These MEMS multimeters could be extremely useful for monitoring applications, including laboratory-based weak electrical signal measurements.

In this paper, we propose a design for a mode-localized sensor based on WCRs. The main characteristic of a mode-localized ECS is the dimensionless output metric of the AR. An input current running through a silicon shunt resistor attached to a sensitive electrode induces stiffness perturbation into WCRs, leading to the redistribution of energy. This means that the input current can be manifested as the change in AR. In this study, the concept of sensing current by mode-localized sensors is demonstrated, and multiple parameters, such as sensitivity, noise floor and bias instability, are tested experimentally.

## Results and discussion

### Working principle of electric current sensors

**Fig. 1:**
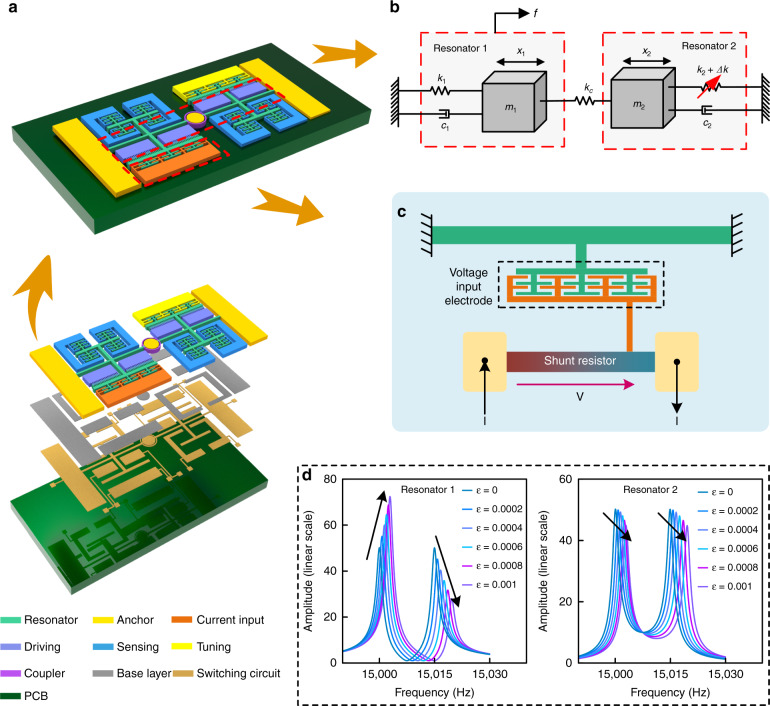
The schematic diagram of the ECS. **a** Structure diagram of ECS**. b** Mass–spring–damper model of WCRs. **c** Structural diagram of the shunt resistor. **d** Amplitude–frequency responses of resonator 1 and resonator 2 versus relative stiffness perturbation ***ε***.

The mode-localized ECS consists of WCRs and shunt resistors. A schematic of the proposed mode-localized ECS is displayed in Fig. 1a. The WCRs consist of two resonators weakly coupled by an annular coupling beam and fixed to both ends along with a central anchor. The shunt resistor is implemented outside the resonators to sense the current.

Resonators are driven by comb finger capacitors. Four sensing electrodes are used to obtain the differential signal, which eliminates feedthrough effects. The external current causes an electrostatic force between the voltage input electrode and the resonator, which is directly transmitted to the resonator as a stiffness perturbation. The perturbation induces the mode localization phenomenon, leading to a significant change in the AR of resonators 1 and 2. Then, the input current is obtained by measuring the variation in the AR. The ECS is fixed on the switching PCB through the base layer and transmits the DC bias voltage, driving the voltage and detection signal through the switching circuit. The design parameters and dimensions are listed in Table 1.

**Table 1:** ECS Parameters.

Parameters	Value
Nominal frequency	14.6 kHz
Quality factor	10,000
Beams of resonators 1 and 2	1820 μm × 20 μm
Shunt resistor	1400 × 30 μm
Gap between capacity	2.5 μm
Comb overlap length	15 μm
Thickness of the device layer	50 μm
Stiffness of resonators 1 and 2	195 N/m
Stiffness of coupling beam	0.2 N/m

### Theoretical analysis of weakly coupled resonator systems

The WCR design can be simplified to a mass–spring–damper model, as shown in Fig. 1b. Resonators in resonant systems vibrate only in the X-direction, and the WCRs are theoretically symmetric. Therefore, the masses and stiffness of the two resonators are designed to be identical. The two resonators are weakly coupled with an annular mechanical beam. The stiffness of the coupling beam is much smaller than that of resonators 1 and 2^32^, with *k*_*c*_ ≪ *k*. The stiffness perturbation $\Delta$*k* caused by the current is much smaller than *k*, with $\Delta$*k* ≪ *k*. With a single resonator driven force *f* and damping coefficient *c*, the motions of the WCRs can be described by differential Eqs.:

1 $$\left\{ {\begin{array}{*{20}{c}} {m\mathop {x}\limits^{..} _1\,\, +\,\, c\mathop {x}\limits^ \cdot _1 + \left( {k + k_c} \right)x_1 - k_cx_2 = f} \\ {m\mathop {x}\limits^{..} _2 \,\, +\,\, c\mathop {x}\limits^ \cdot _2 \,\, +\,\, \left( {k + k_c + {\Delta}k} \right)x_2 - k_cx_1 = 0} \end{array}} \right.$$

According to the Laplace Transform, Eq. (1) can be rewritten as:

2 $$\left[ {\begin{array}{*{20}{c}} {X_1\left( {j\omega } \right)} \\ {X_2\left( {j\omega } \right)} \end{array}} \right] = B^{ - 1}\left[ {\begin{array}{*{20}{c}} {H_{11}} & {H_{12}} \\ {H_{21}} & {H_{22}} \end{array}} \right]\frac{1}{{m\omega _0^2}}\left[ {\begin{array}{*{20}{c}} {F_1\left( {j\omega } \right)} \\ 0 \end{array}} \right]$$

3 $$\left\{ {\begin{array}{lll} {H_{11}\left( {j\omega } \right) = - \frac{{\omega ^2}}{{\omega _0^2}} + j\frac{\omega }{{Q\omega _0}} + 1 + \kappa + \varepsilon } \\ {H_{12}\left( {j\omega } \right) = \kappa } \\ {H_{21}\left( {j\omega } \right) = \kappa } \\ {H_{22}\left( {j\omega } \right) = - \frac{{\omega ^2}}{{\omega _0^2}} + j\frac{\omega }{{Q\omega _0^2}} + 1 + \kappa } \end{array}} \right.$$

4 $$\begin{array}{ll}B = \left( {\frac{\omega }{{\omega _0}}} \right)^4 - \left( {2 + 2\kappa + \varepsilon + \frac{1}{{Q^2}}} \right)\left( {\frac{\omega }{{\omega _0}}} \right)^2 \\\qquad+ 1 + 2\kappa + \varepsilon + \varepsilon \kappa - j\frac{1}{Q}\left( {2\left( {\frac{\omega }{{\omega _0}}} \right)^3 - \left( {2 + 2\kappa + \varepsilon } \right)\frac{\omega }{{\omega _0}}} \right)\end{array}$$

The expressions of *X*_*1*_ and *X*_*2*_ can be deduced from Eq. (2):

5 $$\left\{ {\begin{array}{*{20}{c}} {X_1 = \frac{{\left( { - \frac{{\omega ^2}}{{\omega _0^2}}\,\, +\,\, j\frac{\omega }{{Q\omega _0}}\,\, +\,\, 1\,\, +\,\, \kappa\,\, +\,\, \varepsilon } \right)F_1}}{{Bm\omega _0^2}}} \\ {X_2 = \frac{{\kappa F_1}}{{Bm\omega _0^2}}} \end{array}} \right.$$

According to Eq. (5), the amplitude–frequency responses of two resonators with different input relative stiffness perturbations can be obtained. As shown in Fig. 1d, for resonator 1, the peak values of the 1st mode increase while the peak values of the 2nd mode decrease as the input current increases. For resonator 2, as the input current increases, the 1st and 2nd mode peaks of resonator 2 decrease simultaneously. This result demonstrates that energy transfers from the different modes of the two resonators into the 1st mode of resonator 1^33^, which is one of the features of mode localization.

### Working principle of the shunt resistor

A schematic of the shunt resistor is displayed in Fig. 1c. First, a bias voltage is applied to a resonator. Thereafter, a shunt resistor made of silicon is used to convert the input current into voltage. Subsequently, the potential difference of capacitors between the sensitive electrode and resonator changes, inducing an electrostatic force. The electrostatic force can be written as:

6 $$F_e = \frac{1}{2}\frac{{\varepsilon A\left( {{\Delta}V} \right)^2}}{{\left( {d - x} \right)^2}} = \frac{1}{2}\frac{{\varepsilon A\left( {V_m - I \times R} \right)^2}}{{\left( {d - x} \right)^2}}$$

where *ε* is dielectric constant in vacuum; *A* is the effective area of the resonator and sensitive electrode; $\Delta$*V* is the potential between the resonator and sensitive electrode; *d* is the distance between the two capacitor plates (consisting of a sensitive electrode and a resonator); *x* is the vibration displacement of the resonator; *V*_*m*_ is the bias voltage; *I* is the input current; and *R* is the effective conversion resistance of a shunt resistor made of silicon. *R* can be calculated with the following formula:

7 $$R = \rho \frac{L}{S}$$

where *ρ* is the resistivity of the silicon wafer, which is approximately equal to 0.01 Ω ∙ cm. *L* is the length of the shunt resistor, and *S* represents the cross-sectional area of the shunt resistor. Input electrodes at both ends of the shunt resistor are deposited with Au to reduce the resistance and concentrate it in the shunt resistor. The design parameters of shunt resistance are shown in Table 1. Therefore, the value of the shunt resistor can be calculated to be ~100 Ω.

For WCRs, the fundamental cause of mode localization is the variation in resonator stiffness caused by electrostatic force. The equivalent electrostatic stiffness can be written as:

8 $$k_e = - \left( {{\Delta}V} \right)^2\frac{{\varepsilon A}}{{d^3}} = - \left( {V_m - I \times R} \right)^2\frac{{\varepsilon A}}{{d^3}}$$

### Experimental principle and environment

**Fig. 2:**
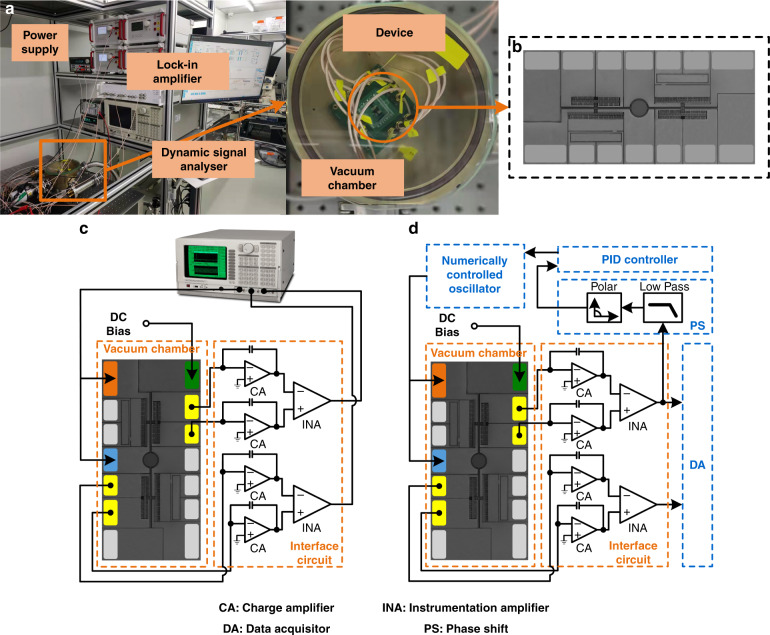
Experimental environment and principle of the ECS. **a** Photo of the experimental environment. **b** SEM photographs of the ECS. **c** Schematic diagram of the open-loop control. **d** Schematic diagram of the closed-loop control.

An experimental schematic of a mode-localized ECS is displayed in Fig. 2. The test is performed in a vacuum chamber at a pressure of 0.086 Pa. The resonators are designed with two outputs to reduce common mode noise. The amplitude signals of resonators are converted, and the differential is amplified by charge amplifiers and instrumentation amplifiers.

The open-loop control principle is outlined in Fig. 2c. A DC of +5 V is applied to the resonators. A 20 mV sweep signal generated by a dynamic signal analyzer (SR785) is placed on the drive ports. The amplitude signals of resonators are converted and differentially amplified by charge amplifiers and instrumentation amplifiers. Afterward, weak output signals from the two outer resonators are observed on the dynamic signal analyzer. Therefore, the mode localization phenomenon can be observed using a dynamic signal analyzer.

A closed-loop control principle is outlined in Fig. 2d. A low-pass filter is used to reduce the high-frequency noise. A Zurich Instrument Phase-locked Amplifier (UHFL-600 MHz) provides a PID controller and numerical oscillator. Considering that the output metric of the WCRs in this study is AR, the output signals of the closed-loop control circuit are sent to a data acquisitor to produce a real-time division.

### Finite element analysis of the electric current sensor

Sensitivity determines the detection limit of the ECS. However, because of the complex structure of the mode-localized ECS, the sensitivity of the ECS cannot be accurately obtained through formula calculations alone under ideal conditions; therefore, it is necessary to run a finite element simulation of the ECS. According to the simulation parameters, COMSOL Multiphysics 5.6 is used to establish a geometric display model. A solid mechanical physical field and a prestressed eigenfrequency analysis step are used for the simulation.

**Fig. 3:**
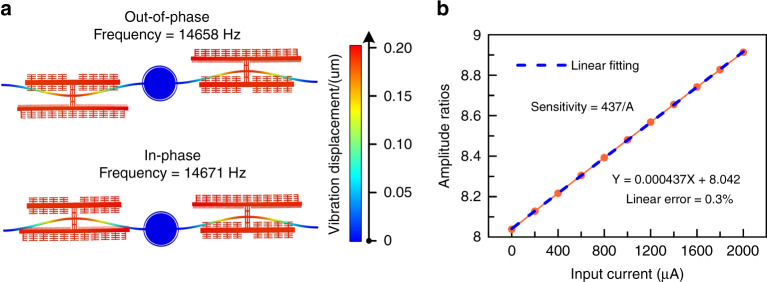
Finite element analysis of WCRs. **a** Resonant frequencies of WCRs. **b** Results of sensitivity simulation.

For WCRs, we design a resonant frequency of ~15 kHz and iterate the structural parameters through simulation. The parameters and dimensions of the ECS are listed in Table 1. The resonant modes are illustrated in Fig. 3a.

In Fig. 3a, the out-of-phase resonant frequency is 14,658 Hz, and the in-phase resonant frequency is 14,671 Hz, with a frequency difference between the two modes of 13 Hz. The amplitudes of the two resonators are almost identical. The frequencies of the two vibration modes are also close, which implies a high sensitivity^34^.

As shown in Fig. 3b, the sensitivity of the ECS reaches 437/A with a 0.3% linear error in a measurement range of 2 mA.

### Vibration mode analysis

**Fig. 4:**
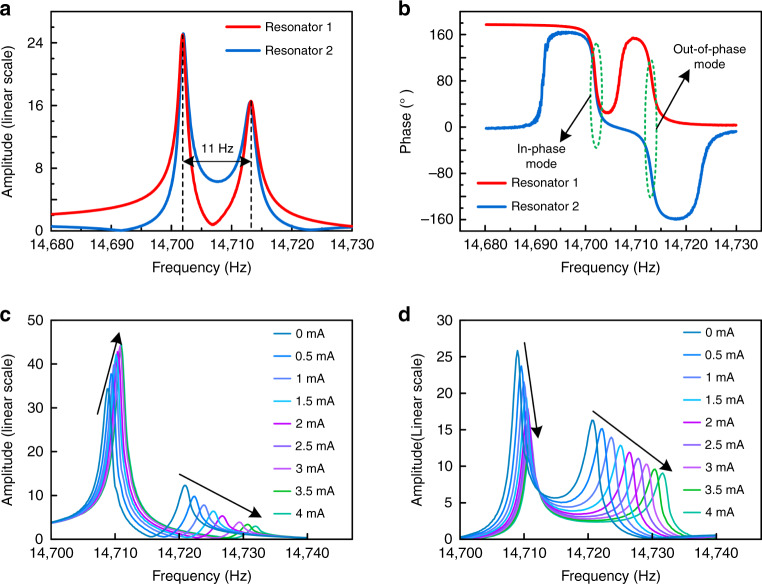
Images of the open-loop test. **a** Amplitude–frequency of resonator 1 and resonator 2. **b** Phase–frequency of resonator 1 and resonator 2. **c** Amplitude–frequency responses of resonator 1 and resonator 2. **d** versus increasing current under open-loop control.

A dynamic signal analyzer provides an AC drive signal for the WCRs and detects amplitude and phase frequency responses. Under open-loop control and without current input, the amplitudes and phases of resonators 1 and 2 on a linear scale are recorded as shown in Fig. 4a, as there are available vibration modes for resonators 1 and 2. The resonant frequencies of the in-phase and out-of-phase modes are 14,702 Hz and 14,713 Hz, respectively, and the frequency difference is 11 Hz. This result is close to the simulation results (Fig. 3a). The initial amplitudes of the two resonators are not equal due to the fabrication tolerance.

According to the phase–frequency response (Fig. 4b), in the 1st mode, the phases of the two modes are identical, but the phase difference between resonators 1 and 2 is 180° in the 2nd mode. The output signals with a 0° phase difference manifest an in-phase vibration mode of the ECS, and 180° indicates an out-of-phase vibration mode. This result is contradictory to the simulation results (Fig. 3a). This is because the sensing electrodes of resonators 1 and 2 are implemented at symmetrical positions; therefore, a 0° phase output signal demonstrates an out-of-phase mode of the ECS. The quality factor of the ECS in the 1st mode is approximately 11,300. After tuning the electrode to compensate for fabrication tolerance, the initial AR is close to the theoretical value of 1.5.

### Frequency response

Under open-loop control, the frequency response of the ECS with different current inputs is tested. As displayed in Fig. 4c, the peak values of the 1st mode increases while the 2nd mode decreases as the input current increases. Resonator 2 shows a variation tendency different from that of resonator 1. As displayed in Fig. 4d, the peak values of both modes decrease as the input current increase. This phenomenon is consistent with the effect previously outlined in Eq. (5) and Fig. 1d. This result demonstrates that energy transfers from the different modes of the two resonators into the 1st mode of resonator 1, thus showing the existence of a mode localization phenomenon.

### Sensitivity measurement

**Fig. 5:**
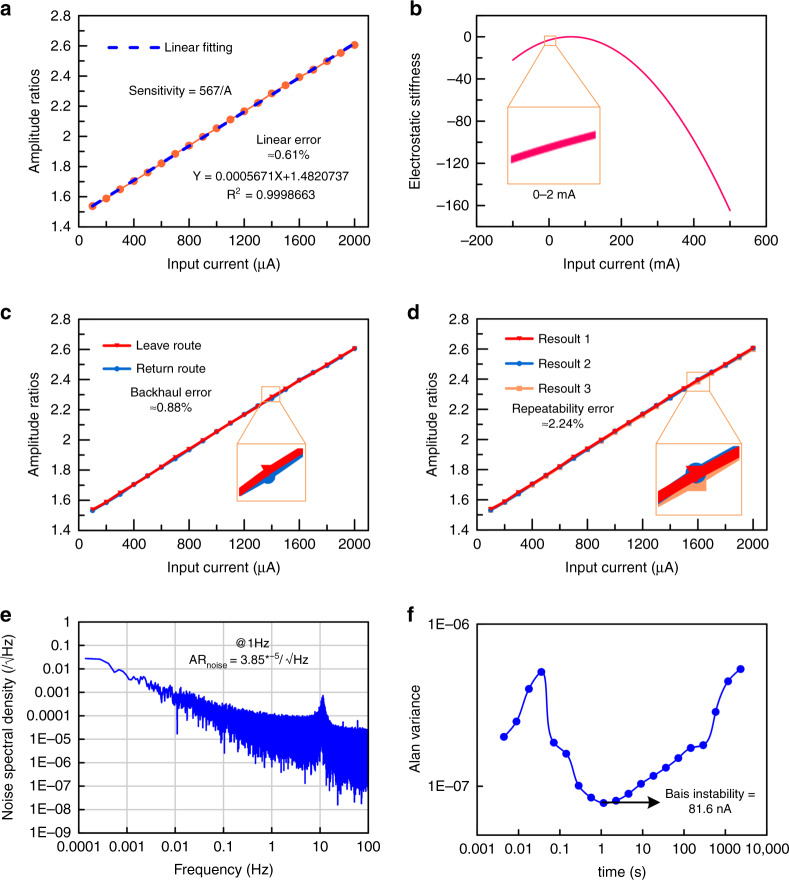
Images of the closed-loop test. **a** AR versus input current. **b** Relation between electrostatic stiffness and input current**. c** Backhaul errors of the ECS**. d** Repeatability errors of the ECS. **e** Noise of resonators 1 and 2. **f** Allan deviation of ECS.

The response of the input current ranging from 0 mA to 2 mA is measured at steps of 200 μA. An adjustment DC voltage of 2.9 V is applied to the tuning electrode on resonator 2 to achieve better linearity and higher sensitivity^23^. Therefore, the initial AR of WCRs is 1.5. As displayed in Fig. 5a, the sensitivity of ECS is measured in closed-loop control. Based on Eq. (8), ECS sensitivity can be adjusted by V_m_, but the higher the sensitivity is, the smaller the measurement range. To compensate for this, V_m_ is selected as 5 V. Figure 5a illustrates the variation in ARs with respect to different currents. The AR increases from 1.5 to 2.6 as the input current ranges from 0 to 2 mA. The measured data are linearly fitted by:

9 $$Y = 0.000567 \ast X + 1.4820737$$

The sensitivity of ECS reaches 567/A at a measurement range of 2 mA. The sensitivity test results of 567/A are different from the simulation results of 437/A. The coupling is thinner because of machining errors, which reduces the coupling stiffness and makes the ECS more sensitive.

### Linearity analysis

According to Eq. (8), the stiffness perturbation is proportional to the square of the voltage difference. Considering that the voltage selected for the WCRs is 5 V, the effective area, distance between the two capacitor plates, and effective conversion resistance are 0.225 mm^2^, 2.5 μm and 82 Ω, respectively. The relationship between the electrostatic stiffness and input current can be calculated as:

10 $$k_e = - 0.127 \times \left( {V_m - 82I} \right)^2$$

As displayed in Fig. 5b, the variation in stiffness is approximately linear when the input current ranges from 0 mA to 2 mA. However, the electrostatic stiffness exhibits a clear, nonlinear behavior when the input current increases. In our design, as displayed in Fig. 5b, the nonlinear error of AR is within a 3% range when the input current varies from 0 to 2 mA. According to the linear fitting curve in Fig. 5a, the linear error can be calculated as follows:

11 $$\sigma _L = \frac{{\left| {{\Delta}L_{{{{\mathrm{max}}}}}} \right|}}{A} = 0.61{{{\mathrm{\% }}}}$$

where $\Delta$*L*_max_ is the largest difference between the measurement and fitting curves, and *A* is the measurement range of the ECS.

The linear error of ECS is less than the error standard of electrical sensing instruments (3%), which ensures the reliability of measurement when the input current changes rapidly.

### Backhaul measurement and repeatability error

As a sensor, the return error and repeatability error must be kept within a reasonable range to ensure measurement accuracy. The input current is measured three times in fronthaul and one time in backhaul.

As shown in Fig. 5c, the backhaul error is calculated as:

12 $$\sigma _h = \frac{{\left| {h_{{{{\mathrm{max}}}}}} \right|}}{A} = 0.88{{{\mathrm{\% }}}}$$

where *h*_*max*_ is the largest difference between the curves of forward travel and reverse travel.

As shown in Fig. 5d, the repeatability error is calculated as:

13 $$\sigma _R = \frac{{3\delta }}{A} = 2.24{{{\mathrm{\% }}}}$$

where *δ* is the largest difference among the five curves for forward and reverse travel.

The backhaul error and repeatability error of the ECS are <3%, which meets the error standard of electrical sensing instruments.

### Noise floor, resolution and dynamic range

A data acquisition card (NUSB6210) is used to record the output signals of resonators 1 and 2 by closed-loop control for two hours with a sample frequency of 225 Hz. Through data processing, two signal amplitude output signals are converted into AR output signals by MATLAB. The output noise of AR is acquired by performing a fast Fourier transform analysis on the acquired data. As displayed in Fig. 5e, the noise power spectral density (PSD) of AR is 3.85 * 10^−5^/√Hz at 1 Hz. The sensitivity of ECS, based on the AR, is 567/A. The noise floor of the ECS can then be calculated as:

14 $$I_{noise} = \frac{{3.85 \ast 10^{ - 5}/\sqrt {Hz} }}{{567/A}} = 67.9\,\,nA/\sqrt {Hz}$$

To measure the resolution of ECS, we test the response time (*t*_*r*_) after current is introduced into the WCRs. The *t*_*r*_ of ECS is approximately 0.23 s. The upper cutoff frequency (*fc*) of ECS is calculated as:^35^

15 $$f_c = \frac{{0.35}}{{t_r}} = 1.5\,\,Hz$$

Because the ECS is a DC current sensor, the lower cutoff frequency (*f*_*l*_) is set to 0.001 Hz. The noise floor *I*_*noise*_ is 67.9 nA/√Hz. The resolution of ECS can be calculated by:^36^

16 $${\Re} s = I_{noise} \times \sqrt {In\left( {\frac{{f_c}}{{f_l}}} \right)} = 183.6\,\,nA$$

The measurement range (*MR*) is 2 mA, and the resolution (*Res*) is 183.6 nA; hence, the dynamic range (*DR*) of the ECS is calculated logarithmically as:

17 $$DR = 20 \times \log \left( {\frac{{MR}}{{{\Re} s}}} \right) = 80.7\,\,dB$$

### Bias instability

Bias instability is an important index for sensors and refers to the random variation in the calculated deviation within a specified finite sampling time and average time interval. According to the effective output signals of the resonators and amplification factor of the circuit, a modified Allan variance of the dataset can be calculated. As displayed in Fig. 5f, the bias instability of the ECS is 81.6 nA.

## Discussion

Compared with traditional electric current sensors, our method has several advantages. First, WCRs are different from the operational amplifier feedback

method, which directly amplifies the current signal to be measured; instead, it amplifies the DC current signal to be measured. The magnitude is modulated to the amplitude of the AC signal, and the measured current signal is amplified by indirect amplification. The WCRs are equivalent to a bandpass filter, which effectively suppresses the noise signal. The method of measuring the size of weak signals by modulation is widely used for weak signal detection purposes.

The performance of ECS and other current sensors is shown in Table 2. Compared with MEMS sensors based on the magnetic principle, the ECS has higher sensitivity due to the principle of mode localization, which increases the detection limit of the ECS. In addition, the ECS is immune to external magnetic fields. Two Fluck 18B+ gears with similar resolution to the ECS are selected for comparison. The resolution of the ECS is higher than that of the μA gear and lower than that of the Sub-μA gear, but the dynamic range of the ECS is larger than either of them, which proves that the ECS has good application prospects. However, the silicon resistor is quite temperature-sensitive, which leads to poor ECS temperature stability. There are two methods to improve this defect in future work. The shunt resistor can be made from temperature-insensitive materials by a deposition process, which greatly enhances the resistance of the ECS to temperature changes. In addition, optimizing the design to increase the resistance of the shunt resistor would be a good approach. According to Eqs. (8), (14) and (16), the ECS resolution can be improved by increasing the resistance of the shunt resistor. The heat produced by the shunt resistor is proportional to the resistance and the square of the current. Therefore, improving the resolution of the ECS can effectively reduce the thermal effect of the current.

**Table 2:** Comparison of Performance Indices of Different Current Sensors.

Performance indices	ECS	Magnetic metal film^5^	Giant magnetoresistance^6^	Fluke 17B+ (Sub-μA gear)	Fluke 17B+ (μA gear)
Sensitivity	567/A	5 √Hz/A	22 V/A	–	–
Resolution	183.6 nA	–	Sub-mA	100 nA	1 μA
Measurement range	2 mA	1.5 A	2 mA	400 μA	4 mA
Dynamic range	80.7 dB	–	–	72 dB	72 dB

## Conclusion

In this study, a mode-localized ECS based on a WCR is proposed and experimentally demonstrated for the first time. With the advantage of high-voltage sensitivity of WCRs, current is converted to voltage through a sampling resistor made of silicon, causing a stiffness perturbation to one outer resonator. This alters the energy distribution in the WCRs because of the mode localization phenomenon. The sensitivity of the ECS is 567/A, the noise floor is 67.9 nA/√Hz, the resolution is 183.6 nA, and other performance indices are also tested.

However, the sensitivity, noise floor, resolution and dynamic range of this sensor should be further improved for wider applications. Optimizing the design to increase resistance and crafting sensor arrays would be good ways to improve current detection. In addition, there is a possibility of combining the ECS and mode-localized voltmeter to create a MEMS multimeter in the future.

## Materials and fabrication

**Fig. 6:**
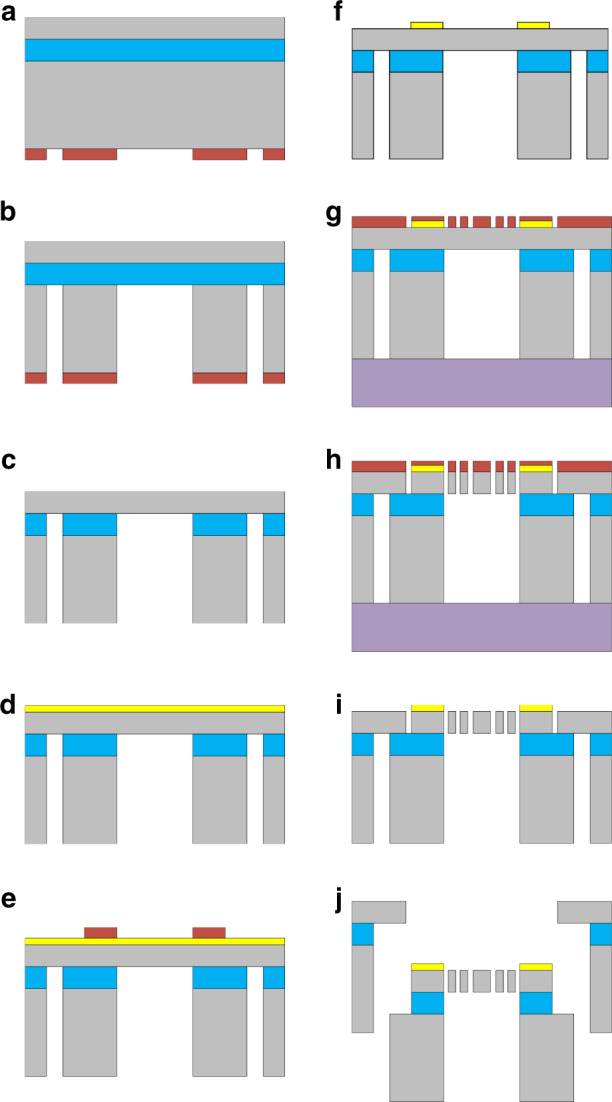
Fabrication process of the ECS. **a** Processes for patterning the bottom layer. **b** ICP etching of the basal layer. **c**, **d** Removal of substrate photoresist and deposition of Au. **e** Processes for patterning the metal layer. **f** Metal corrosion and photoresist removal of metal layers. **g** Processes for patterning the structure layer and pasting the support chip. **h** ICP etching of the structural layer. **i** Removal of photoresist and support chip. **j** Chip release.

The ECS was fabricated using a dicing-free silicon-on-insulator (SOI)^37^ process with a 50 μm thick device layer (as illustrated in Fig. 6).

The back cavity was made first. The back cavity reduced the release time of the last step and effectively reduced the influence of the footing effect on the ECS

structure. Because the thickness of the base layer reached 450 µm, AZ 4620 was selected as a positive photoresist for patterning the bottom layer. AZ 4620 reached ~7–8 µm. Subsequently, the silicon wafer was placed into an ICP etching chamber for etching, which reached as far as the middle oxide layer. The silicon wafer was then placed in an acetone solution for 30 min to dissolve the photoresist.

Second, EI 1680 was selected for patterning the metal layer with a thickness of ~2.5 µm. Subsequently, Au was used as the electrode material. The thickness of the deposited Au was 150 nm. The removal of Au with a lateral corrosion error should be <5 μm.

EI 1680 was then selected for patterning the structure layer with a thickness of ~3 µm, and the silicon wafer was bonded to the supporting wafer. Two points were considered when bonding the silicon and the supporting wafer: first, the pressure on both should be uniform to prevent any gap between the silicon and the supporting wafer from affecting the thermal conductivity of the whole bond sheet; second, the pressure between the two was maintained for a certain period of time until the photoresist was dry to ensure bond strength. Thereafter, the silicon wafer was etched into the ICP etching chamber to etch the middle oxide layer. Figure 6h shows the silicon chip after etching of the grooves and chambers in the base layer. At this time, the ECS chip and wafer frame were physically separated on the base layer. After that, the bonded wafers were immersed in acetone solution for 30 min to dissolve the photoresist, allowing the silicon wafers to be separated from the supporting wafers.

Finally, self-separation by the box layer was released. Released holes were implemented on the support, which was on the outside of the ECS. The device and support were self-separated after the final step of the box layer release.

## References

[1] Barrell H (1961). Eleventh general conference of weights and measures. Nature.

[2] Zhou C-Y (2012). An accurate low current measurement circuit for heavy iron beam current monitor. Nucl. Instrum. Methods Phys. Res. Sect. B-Beam Interact. Mater. At..

[3] Ding L, Ma C, Li L, Zhang L, Yu J (2016). A photoelectrochemical sensor for hydrogen sulfide in cancer cells based on the covalently and in situ grafting of CdS nanoparticles onto TiO2 nanotubes. J. Electroanalytical Chem..

[4] Popovic RS (2000). Not-plate-like Hall magnetic sensors and their applications. Sens. Actuators a-Phys..

[5] Tuan Anh P, Hara M, Oguchi H, Kuwano H (2015). Current sensors using Fe-B-Nd-Nb magnetic metallic glass micro-cantilevers. Microelectron. Eng..

[6] Garcia-Romeo, D. et al. Sub-mA current measurement by means of GMR sensors and state of the art lock-in amplifiers. *IEEE International Conference on Industrial Technology (ICIT)*. 3377–3381 (2015).

[7] Giesen, F., Podbielski, J. & Grundler, D. Mode localization transition in ferromagnetic microscopic rings. *Phys. Rev. B* **76**, 10.1103/PhysRevB.76.014431 (2007).

[8] Hodges CH (1982). Confinement of vibration by structural irregularity. J. Sound Vib..

[9] Hodges CH, Woodhouse J (1986). Theories of noise and vibration transmission in complex structures. Rep. Prog. Phys..

[10] Hodges CH, Woodhouse J (1989). Confinement of vibration by one-dimensional disorder .1; Theory of ensemble averaging. J. Sound Vib..

[11] Pierre C (1988). Mode localization and eigenvalue loci veering phenomena in disordered structures. J. Sound Vib..

[12] Pierre C, Tang DM, Dowell EH (1987). Localized vibrations of disordered multispan beams-theory and experiment. Aiaa J..

[13] Anderson PW (1958). Absence of diffusion in certain random lattices. Phys. Rev..

[14] Pandit M, Zhao C, Sobreviela G, Seshia A (2020). Practical limits to common mode rejection in mode localized weakly coupled resonators. Ieee Sens. J..

[15] Zhong, J., Yang, J., Chang, H. & Ieee. The temperature drift suppression of mode-localized resonant sensors. *31st IEEE International Conference on Micro Electro Mechanical Systems (MEMS)*. 467–470 (2018).

[16] Zhang, H., Zhong, J., Yuan, W., Yang, J. & Chang, H. Ambient pressure drift rejection of mode-localized resonant sensors. 30th *IEEE International Conference on Micro Electro Mechanical Systems (MEMS)*. 1095–1098, 10.1109/MEMSYS.2017.7863604 (2017).

[17] Thiruvenkatanathan P, Yan J, Seshia AA (2010). Differential amplification of structural perturbations in weakly coupled MEMS resonators. Ieee Trans. Ultrason. Ferroelectr. Frequency Control.

[18] Spletzer, M., Raman, A., Wu, A. Q., Xu, X. & Reifenberger, R. Ultrasensitive mass sensing using mode localization in coupled microcantilevers. *Appl. Phys. Lett.* **88**, 10.1063/1.2216889 (2006).

[19] Gil-Santos E (2009). Mass sensing based on deterministic and stochastic responses of elastically coupled nanocantilevers. Nano Lett..

[20] Spletzer, M., Raman, A., Sumali, H. & Sullivan, J. P. Highly sensitive mass detection and identification using vibration localization in coupled microcantilever arrays. *Appl. Phys. Lett.* **92**, 10.1063/1.2899634 (2008).

[21] Thiruvenkatanathan, P., Yan, J., Woodhouse, J., Aziz, A. & Seshia, A. A. Ultrasensitive mode-localized mass sensor with electrically tunable parametric sensitivity. *Appl. Phys. Lett.* **96**, 10.1063/1.3315877 (2010).

[22] Zhang H, Li B, Yuan W, Kraft M, Chang H (2016). An acceleration sensing method based on the mode localization of weakly coupled resonators. J. Microelectromechanical Syst..

[23] Zhang, H., Sobreviela, G., Chen, D., Pandit, M. N. & Seshia, A. A High-performance mode-localized accelerometer employing a quasi-rigid coupler. *IEEE Electron Device Letters*, 1–1 (2020).

[24] Zhang H (2022). Mode-localized accelerometer in the nonlinear Duffing regime with 75 ng bias instability and 95 ng/√Hz noise floor. Microsyst. Nanoengineering.

[25] Manav, M., Reynen, G., Sharma, M., Cretu, E. & Phani, A. S. Ultrasensitive resonant MEMS transducers with tuneable coupling. *J Micromech Microeng.* **24**, 10.1088/0960-1317/24/5/055005 (2014).

[26] Thiruvenkatanathan, P., Yan, J., Seshia, A. A. & Ieee. Ultrasensitive Mode-Localized Micromechanical Electrometer. *2010 IEEE International Frequency Control Symposium*. 91–96 (2010).

[27] Zhang, H. et al. A High-sensitive resonant electrometer based on mode localization of the weakly coupled resonators. *29th IEEE International Conference on Micro Electro Mechanical Systems (MEMS)*. 87–90 (2016).

[28] Van, Z., Liang, J., Hao, Y., Chang, H. & Ieee. A micro resonant dc electric field sensor based on mode localization phenomenon. *32nd IEEE International Conference on Micro Electro Mechanical Systems (IEEE MEMS)*. 849–852 (2019).

[29] Zhao C (2016). A Three Degree-of-Freedom weakly coupled resonator sensor with enhanced stiffness sensitivity. J. Microelectromech. Syst..

[30] Hao Y, Liang J, Kang H, Yuan W, Chang H (2021). A micromechanical mode-localized voltmeter. Ieee Sens. J..

[31] Li, W. et al. A mode-localized magnetometer with resolution of 6.9 nT/root hz within the range of 100 mT. *33rd IEEE International Conference on Micro Electro Mechanical Systems (MEMS)*. 190–193 (2020).

[32] Zhao C (2016). A review on coupled MEMS resonators for sensing applications utilizing mode localization. Sens. Actuators a-Phys..

[33] Zhang H, Chang H, Yuan W (2017). Characterization of forced localization of disordered weakly coupled micromechanical resonators. Microsyst. Nanoeng..

[34] Zhang H (2021). A low-noise high-order mode-localized mems accelerometer. J. Microelectromech. Syst..

[35] Bogatin, E. Rule of Thumb #1: Bandwidth of a signal from its rise time.

[36] Instruments, T. Noise analysis in operational amplifier circuits. Application Report, SLVA043B (2007).

[37] Hao Y, Xie J, Yuan W, Chang H (2016). Dicing-free SOI process based on wet release technology. Micro Nano Lett..

